# Supplementary material for: The genome sequence of the Currant Shoot Borer, Lampronia capitella (Clerck, 1759) (Lepidoptera: Prodoxidae)
Source: Wellcome Open Res. 2026 Oct 30;11:946. [Version 1] doi: 10.12688/wellcomeopenres.28270.1 (PMC13770172; doi:10.12688/wellcomeopenres.28270.1)
Supplement: Peer review history

# Reviewer report: Reviewer response for version 1

**DOI:** 10.21956/wellcomeopenres.31098.r177596

**Published:** 2026-11-17

**Authors:** Jonathan Badger

**Affiliations:** 1 Laboratory of Integrative Cancer Immunology, National Cancer Institute Center for Cancer Research, Bethesda, Maryland, USA

The authors describe their sequencing of an individual female shoot borer of the species *Lampronia capitella*. The assembly was of reasonably high quality, and the Z sex chromosome was found among the scaffolds. The sequencing was done using long-read PacBio technology and the assembly and analysis was performed as is standard in the Tree of Life genome project.

Are the datasets clearly presented in a usable and accessible format, and the assembly and annotation available in an appropriate subject-specific repository?

Yes

Are sufficient details of the sequencing and extraction, software used, and materials provided to allow replication by others?

Yes

Are the rationale for sequencing the genome and the species significance clearly described?

Yes

Are the protocols appropriate and is the work technically sound?

Yes

Reviewer Expertise:

I am a computational genomicist, currently working in the biomedical field but who has also participated in insect genome projects.

I confirm that I have read this submission and believe that I have an appropriate level of expertise to confirm that it is of an acceptable scientific standard.

# Reviewer report: Reviewer response for version 1

**DOI:** 10.21956/wellcomeopenres.31098.r179178

**Published:** 2026-11-14

**Authors:** Bengt Hansson

**Affiliations:** 1 Lund University, Lund, Sweden

This genome note presents a high-quality, chromosome-level genome assembly for the Currant Shoot Borer, *Lampronia capitella*, generated as part of the Darwin Tree of Life project. The authors provide assemblies for two haplotypes, with most of haplotype 1 assigned to 31 chromosomal pseudomolecules, including the Z chromosome. The mitochondrial genome is also assembled. Overall, this represents a valuable genomic resource for *L. capitella* and adds to the growing genomic resources available for Lepidoptera.

 The manuscript is clear and appropriately concise for a genome assembly note. The assembly appears to be of high quality, and the resource will be useful for future comparative, evolutionary and population genomic studies.

Are the datasets clearly presented in a usable and accessible format, and the assembly and annotation available in an appropriate subject-specific repository?

Yes

Are sufficient details of the sequencing and extraction, software used, and materials provided to allow replication by others?

Yes

Are the rationale for sequencing the genome and the species significance clearly described?

Yes

Are the protocols appropriate and is the work technically sound?

Yes

Reviewer Expertise:

Evolutionary biology, population genetics, comparative genomics

I confirm that I have read this submission and believe that I have an appropriate level of expertise to confirm that it is of an acceptable scientific standard.

# Reviewer report: Reviewer response for version 1

**Published:** 2026-10-30

**Authors:** Automated Benchmarking

**Affiliations:** 1 Wellcome Sanger Institute, Hinxton, UK

As this article is a Genome Note, Automated Benchmarking has been used to determine whether the reported assembly passes four defined checks established by the Earth BioGenome Project: the raw and assembled data are publicly available, the Earth BioGenome Project three-part metric is met, the k-mer completeness exceeds 90%, and the BUSCO score passes the stated threshold. For Lampronia capitella (ilLamCapi1), all four checks passed. Data available: pass. ENA study PRJEB100927, raw-read accessions ERR15743641 and ERR15749349, and assembly accessions GCA_977035995.1 and GCA_977035765.1 are public. Earth BioGenome Project three-part metric: pass. Haplotype 1 is reported as 6.C.Q65, meeting the 6.C.Q40 reference standard. k-mer completeness: pass. The combined haplotype assemblies have 99.63% k-mer completeness, exceeding the Earth BioGenome Project target of 90%. BUSCO: pass. Haplotype 1, which is proposed as the reference assembly for this species, has 94.0% complete BUSCOs (93.4% single-copy and 0.6% duplicated). BUSCO software version: 6.1.0; lineage dataset: lepidoptera_odb12. It meets the target of greater than 90% complete and single-copy BUSCOs. This decision uses values reported in the Genome Note and public accession records.

Reviewer Expertise:

NA

I confirm that I have read this submission and believe that I have an appropriate level of expertise to confirm that it is of an acceptable scientific standard.
